# A psychometric evaluation of the Italian short version of the Fear of Pain Questionnaire-III: Psychometric properties, measurement invariance across gender, convergent, and discriminant validity

**DOI:** 10.3389/fpsyg.2022.1087055

**Published:** 2023-01-11

**Authors:** Pierluigi Diotaiuti, Stefano Corrado, Stefania Mancone, Elisa Cavicchiolo, Andrea Chirico, Thais Cristina Siqueira, Alexandro Andrade

**Affiliations:** ^1^Department of Human Sciences, Society and Health, University of Cassino and Lazio, Cassino, Italy; ^2^Department of Systems Medicine, Tor Vergata University of Rome, Rome, Italy; ^3^Department of Psychology of Development and Socialization Processes, Sapienza University of Rome, Rome, Italy; ^4^Health and Sports Science Center, Department of Physical Education, CEFID, Santa Catarina State University, Florianopolis, Santa Catarina, Brazil

**Keywords:** fear, pain, FPQ-III, psychometrics, catastrophizing, anxiety, gender, non-clinical sample

## Abstract

**Introduction:**

The *Fear of Pain Questionnaire-III* (FPQ-III) is a self-assessment instrument developed specifically to measure fear based on various pain stimuli converging on three factors: severe pain, medical pain, and minor pain. It actually remains the most studied and internationally used tool even in its short versions. The aim of this work was to propose a new validation study oriented to confirm the good psychometric properties of a short model of the FPQ-III for the Italian context.

**Methods:**

A large sample of participants was recruited (*n*  = 1,064) and Exploratory Factor Analysis (EFA) as well as Confirmatory Factor Analysis (CFA) were performed. Measurement invariance of the FPQ-III across gender was also evaluated. In order to examine convergent validity, a further convenient sample (*n*  = 292) was used and variables related to the individual’s pain experience, locus of control and coping orientations were assessed. A final discriminant assessment using experimental manipulation through fear eliciting videos was performed.

**Results:**

The three factors structure of the 13-item version of the questionnaire was confirmed (χ^2^ = 148.092, CFI = 0.971, TLI = 0.962, RMSEA = 0.046, RMSEA 90% CI = 0.037–0.056) as well as the measurement invariance across gender. Item internal reliability was satisfactory. The results provided evidence of the good predictive validity of the FPQ-III and the discriminant assessment demonstrated that the instrument is suitable in detecting changes in fear of pain induced by specific situational conditions.

**Discussion:**

The scale in this short version is suitable for quickly and efficiently gathering information about the perceived intensity of such anticipatory fears that might affect even the healthy person dysfunctionally.

## Introduction

Fear is defined as an emotional reaction to a specific, identifiable and immediate threat (McNeil and Vowles, 2004). When it is related to pain, it can evolve as a result of negative interpretations and catastrophic amplifications according to which pain is judged as equivalent to ongoing harm (Turk and Wilson, 2010). Fear falls under the primary control of the amygdala, described as the emotional center of the brain (Neugebauer, 2015). This brain area intervenes in the emotional physiological response, playing a primary role in emotional regulation following a painful stimulus, and on its related modulation (Öhman, 2005; Hulsman et al., 2021; Šimić et al., 2021). The model proposed by Vlaeyen and Linton (2000) posits that there may be two types of opposing behavioral responses: confrontation and avoidance of pain (Linton et al., 2018). They conducted an extensive analysis on the fear-avoidance model, highlighting possible precursors of pain-related fear, including the role of anxiety about pain as a limiting element to functional abilities (Lucchetti et al., 2012).

In the past it has been stated that confrontation leads to a reduction or elimination of fear, while avoidance maintains and increases fear to the point of becoming a phobia (Lethem et al., 1983; Philips and Jahanshahi, 1986). By analyzing these two responses, several factors can be associated that may go to influence this process such as negative evaluations regarding pain, avoidance and escape behavior, or hypervigilance (Leeuw et al., 2007). Some findings indicate that one of the reasons why individuals have a high fear of pain is because they are particularly susceptible to negative experiences of pain (Keogh et al., 2001).

According to current fear-avoidance models, when pain is perceived, judgments are made about the purpose of that pain (Crombez et al., 2012) and some attribute catastrophic meaning to pain, which, in turn, elicits fear of pain (Goubert et al., 2004). Since fear has a significant bearing on pain, the assessment of fear of pain turns out to be an important task for scholars to understand the mechanisms and individual differences in the very role of anxiety and fear (Ochsner et al., 2006). However, the relationship between avoidance behavior and the individual’s specific fears is very complex as it is difficult to establish the actual relationship. In this regard, McNeil and Rainwater (1998) developed the *Fear of Pain Questionnaire* (FPQ) with the aim of detecting by means of a questionnaire the excessive fear of pain both in individuals with chronic pain and in nonclinical individuals (Osman et al., 2002).

The most recent form of the questionnaire is the *Fear of Pain Questionnaire-III* (FPQ-III), a self-assessment instrument developed specifically to assess fear based on various pain stimuli. The FPQ-III includes 30 items from which three subscales (Severe Pain, Minor Pain, and Medical Pain) can be measured. Although many studies have evaluated its validity and reliability (Carleton and Asmundson, 2009; Yang et al., 2013), other studies have identified limitations in the instrument, confirmed by the same factor analysis, indicating that improvements could be applied to the instrument itself (Roelofs et al., 2005). Albaret et al. (2004) conducted a study to examine the factorial structure of the Fear of Pain questionnaire on three samples consisting of young, adult and elderly Europeans. The authors, in addition to identifying the French-language adaptation of the questionnaire, tested the inverse relationship between previous exposure to pain and fear of pain, noting significant differences in model fit based on the use of shorter versions with 15 items of the FPQ-III questionnaire. However, there are other instruments in the literature, which similar to the FPQ measure constructs associated with fear of pain. These include the *Pain Anxiety Symptoms Scale* (McCracken et al., 1992), the *Fear Avoidance Beliefs Questionnaire* (FABQ, Waddell et al., 1993; Pfingsten et al., 2000); the *Dental Fear Assessment Scale* (DFAS, Rowe, 1997), the *Fear-Avoidance of Pain Scale* (Crowley and Kendall, 1999), the *Brief Behavioral Distress Scale* (BBDS, Tucker et al., 2001), the *Child Adult Medical Procedure Interaction Scale* (CAMPISSF, Blount et al., 2001).

By the way, McNeil and Rainwater’s (1998) instrument remains the most studied and used internationally, even in its short versions such as the FPQ-Short Form consisting of 20 items (FPQ-SF) (Asmundson et al., 2008) and the FPQ-9 consisting of 9 items (McNeil et al., 2018).

Some research that has employed this questionnaire has identified gender differences on fear of pain, that confirms women’s tendency to react more fearfully to painful stimuli (Thibodeau et al., 2013; Vambheim and Øien, 2017). Other scholars have pointed out that cultural and linguistic factors may also play an important role in the assessment of fear of pain (Orhan et al., 2018).

In the international context, Spanish (Solé et al., 2019), French (Albaret et al., 2004), Dutch (Van Wijk and Hoogstraten, 2006), Portuguese (Cardoso et al., 2016), and Norwegian (Vambheim et al., 2017) validations are currently available. Di Tella et al. (2019) first proposed an Italian study of the FPQ-III, comparing the 30-item version with the short 20-item and 9-item versions. They employed a heterogeneous sample of Italian adults and showed the psychometric properties of the three models exhibited acceptable fit values overall, however better in the short versions of the instrument. They reported divergent validity with anxiety and depression measures, test–retest reliability, and measurement invariance across gender and different age groups.

The purpose of our work is to propose a new validation study oriented to confirm the good psychometric properties of a short version of the FPQ-III for the Italian context. Since among the limitations of the study by Di Tella et al. (2019) was mentioned the disproportion in the gender distribution among participants, in order to be able to exhibit robust results with respect to the measurement invariance, in our survey we recruited a large number of participants with substantially homogeneous distribution with respect to gender. To assess the convergent validity of the instrument, we combined the administration of the short version with three additional measures, different from the two used in the previous study by Di Tella et al. (2019) that were related to anxiety and depression: the *Pain Catastrophing Scale* (PCS, Sullivan et al., 1995; Italian validation Monticone et al., 2012), the *Locus of Control of Behavior Scale* (LCBS, Craig et al., 1984; Italian validation Farma and Cortivonis, 2000), and the *Coping Orientations to Problems Experienced* (COPE, Carver et al., 1989; Italian validation COPE-NVI, Sica et al., 2008). Considering the literature evidence, we therefore hypothesized to find significant associations with PCS, both total but particularly with the brooding subscale (Burri et al., 2018; Priore et al., 2019; Diotaiuti et al., 2021; Nunziato et al., 2021), with the external locus of control of the LBCS (Seville and Robinson, 2000; Torres et al., 2009; Sweeney et al., 2018), with the avoidance coping style (Benoit-Piau et al., 2018; Du et al., 2018; Ibrahim et al., 2020; Lentz et al., 2022) and transcendent reliance strategies of the COPE-NVI (Flanigan et al., 2019; Ferreira-Valente et al., 2020).

In order to lend support to the discriminant validity of the instrument, we added an additional laboratory study using three short 20-s videos as pain fear manipulation tools with the aim of eliciting by their presentation to participants an internal activation with respect to the sources of severe, medical, and minor pain. We hypothesized that with respect to a control group, the measurement using the FPQ-III short form would have revealed, in the comparison before and after the presentation of the videos, a significant increase in the scores related to the subscales of fear of severe, medical, or minor pain, confirming that the scale can adequately be used for both dispositional measurement of fear of pain but also for the assessment of a state condition.

## Materials and methods

### Linguistic procedures

According to the EORTC translation rules, the FPQ-III was translated using forward and backward translations of the original scale (Dewolf et al., 2009). Two Italian translators completed the forward translation independently and worked out any inconsistencies between the two versions. Two English translators separately back-translated the measure after receiving the reconciled Italian version. Any differences were discussed and resolved, and changes were made to the FPQ-III to account for any rewording in order to improve the items’ conceptual relevance and comprehension. Finally, a small focus group of 10 people was formed and constructed to include people from three different age groups (20–30; 31–40; 41–50), both genders, and individuals with low, medium, and high educational qualifications. Following the administration of the FPQ-III scale, a discussion of each item revealed no issues of comprehensibility or literacy disparities.

### Participants and administration

The sample size for this study was determined by the ability to demonstrate a satisfactory fit of FPQ-III, which began with a translation of the full English questionnaire, which contained a three-factor model with 30 manifest variables. Using the root-mean-square error of approximation (RMSEA) as the measure of model fit, a minimum of 300 participants provides a 90% power level to test RMSEA **≤** 0.05 when RMSEA = 0.08, using a 0.05 significance level (MacCallum et al., 1996). Participants were recruited by sending contact to university students in central Italy, outlining the study’s goals and purpose. Participants were instructed to click on a URL provided in the same notice, fill out the form, and then telematically and digitally submit their responses. Participants were guaranteed anonymity as well as the usage of aggregate data for research purposes. A total of 3,400 emails with contact information were sent. In terms of the drop-out rate, 114 people dropped out after starting to fill it out, resulting in a total of 708 completed questionnaires (333 males and 375 females with an average age of 22.67 and *SD* = 2.47). The convergent validity was examined using an additional convenient sample of 292 people, 113 males (38.7%) and 179 females (61.3%), M_age_ 24.46, and *SD* = 7.39, all of whom were recruited online. The non-participation in the previous administration was the inclusion requirement in this case. Concerning the assessment of the discriminant validity of the instrument, which was referred to at the end of the introduction, and which involved repeated measures of the FPQ-III short version interspersed with the viewing of videos fear of pain according to the classification of the scale (severe, minor, medical), a design for a two-way repeated measures ANOVA with two independent variables (group, video) and one dependent variable (fear of pain) with four groups was prepared. The design consists of three experimental groups, which were administered a video elicitating fear of severe pain, a video elicitating medical fear, a video elicitating fear of minor pain, and a control group, which only took the two scale measurements without the variable manipulation stimulus, respectively. The number of participants was defined through the use of the statistical software G-Power calculator in the minimum number of 56 participants. Therefore, following an invitation for participation in the study open to college students, 64 individuals (32 males, M_age_ = 22.47) who indicated their willingness to participate in the study were recruited. Again, the inclusion criterion was to have not participated in previous administrations of the study. They have been assigned to the four groups by a randomized distribution that, however, balanced in the groups the gender variable (8 males and 8 females). Pre- and post-viewing of the videos were administered in the laboratory through a computerized administration procedure and with a 24-h interval. The whole recruitment process was conducted in the months of January and March 2022.

The selection of videos to be shown to the participants was made preliminarily through the involvement of an *ad hoc* pool consisting of 24 students (14 females and 10 males) who were asked to judge the relevance and emotional solicitation stimulated by viewing a series of 27 short copyright-free videos, collected from the Internet, that showed content assimilated to the categories of medical pain, severe pain and minor pain. The videos were first ascribed to one of three categories and prioritized with respect to the strength of suggestion/stimulation produced using a rating on a scale of 1 to 10 (Alivernini et al., 2008). A further selection was made by asking students to eliminate from the three groups those videos that might present content perceived as too strong or disturbing to the participant. At this point, considering the hierarchical order of classification, the first three videos in each category were deemed useful for use in the study, which were then edited to form three separate clips with a total duration of 50 s. In the first, representative of medical pain, there were a scene depicting an infiltration puncture to the knee, a scene of a suturing of the superior labrum, and a scene of an orthopedic traction of a fractured leg; in the second, representative of severe pain, there were a scene of a fall from a horse, a scene depicting a muscle injury at the gym, and a scene referring to a bicycle accident on the road; in the third, representative of mild pain, there were a scene of an accidental hammering on the hand, a scene of a barefoot stomping on a sharp object, and a scene depicting the execution of an axillary depilatory waxing. All participants gave written informed consent to participate in this study. The protocol was approved by the local university Institutional Review Board.

### Measures

- *Fear of Pain Questionnaire* (FPQ-III, McNeil and Rainwater, 1998) is a 30-item self-report measure of pain-related fear designed to tap fear related to severe pain (e.g., “Breaking your leg”), minor pain (e.g., “Getting a paper-cut on your finger”), and medical pain (e.g., “Receiving an injection in your hip/buttocks”). Each item seeks to represent a potentially painful relatively common and accessible to individual’s experience, even if indirectly, by sharing experiences with others. Items are scored on a 5-point scale ranging from 1 (not at all) to 5 (extreme). The overall score (range 30 to 150) and subscale scores (range 10 to 50) will be calculated for every participant. The higher the score obtained (range 30–150), the greater the pain of fear levels. The original study reported good internal consistency (ω = 0.92 for total scale; ω = 0.88 for severe pain; ω = 0.87 for minor pain; ω = 0.92 for medical pain) and good test–retest reliability (ω = 0.74 for total scale; ω = 0.69 for severe pain; ω = 0.73 for minor pain; ω = 0.76 for medical pain).
- The *Pain Catastrophizing Scale* (PCS, Sullivan et al., 1995; Italian validation Monticone et al., 2012) was developed to help quantifying individuals’ pain experience with questions about how they feel and what they think about when they are in pain. The PCS consists of 13 items with a score assigned on a scale from 0 (never) to 4 (always) measuring three different variables: rumination, magnification (“amplification”), and sense of powerlessness (“helplessness”). Pain catastrophizing is characterized by a tendency to increase the threat value of a pain stimulus and to feel helpless in the presence of pain, as well as a relative inability to prevent or inhibit pain-related thoughts in anticipation of, during or after a painful event. Higher scores indicate higher levels of pain-related anxiety. The following pain anxiety severity levels are recommended for clinical interpretation: mild = 0 to 34; moderate = 35 to 67; and severe = 68 to 100. Reliability measures for this study: Rumination α = 0.80 [CIs 95% 0.786; 0.850], ω = 0.82; [CIs 95% 0.791; 0.855]; Amplification α = 0.66 [CIs 95% 0.561; 0.701], ω = 0.67; [CIs 95% 0.565; 0.704]; Helplessness α = 0.84 [CIs 95% 0.814; 0.869], ω = 0.85; [CIs 95% 0.823; 0.875]; Total score α = 0.90 [CIs 95% 0.887; 0.919], ω = 0.90; [CIs 95% 0.888; 0.920].
- The *Locus of Control of Behavior Scale* (LCBS, Craig et al., 1984; Italian validation Farma and Cortivonis, 2000), is a self-report instrument comprising 17 items and it measures the internal (e.g., “I can anticipate difficulties and take action to avoid them”) and external (e.g., “my life is controlled by outside actions and events”) locus of control. The items are rated on a six-point Likert scale, ranging from 0 (strongly disagree) to 5 (strongly agree), with higher scores representing a higher level of externality. Reliability for this study: Internal locus α = 0.70 [CIs 95% 0.641; 0.733]; ω = 0.70; [CIs 95% 0.643; 0.730]; External locus α = 0.73 [CIs 95% 0.679; 0.772]; ω = 0.73; [CIs 95% 0.683; 0.774].
- The *Coping Orientations to Problems Experienced* (COPE, Carver et al., 1989; Italian validation COPE-NVI, Sica et al., 2008) is composed of five major independent dimensions (social support, avoidance, positive attitude, problem orientation and transcendent orientation). Its 60 questions investigate the ways in which people react to stressful events. For each of the statements, people are asked to score how often they use that particular “strategy” *via* a 4-point scale. Reliability measures for this study: Social support α = 0.89 [CIs 95% 0.871; 0.908], ω = 0.89; [CIs 95% 0.873; 0.908]; Avoidance α = 0.79 [CIs 95% 0.752; 0.822], ω = 0.72; [CIs 95% 0.651; 0.785]; Positive attitude α = 0.79 [CIs 95% 0.753; 0.822], ω = 0.79; [CIs 95% 0.752; 0.823]; Problem orientation α = 0.83 [CIs 95% 0.794; 0.852], ω = 0.83; [CIs 95% 0.799; 0.857]; Transcendent orientation α = 0.81 [CIs 95% 0.768; 0.838], ω = 0.80; [CIs 95% 0.763; 0.830]; Total score α = 0.86 [CIs 95% 0.832; 0.878], ω = 0.83; [CIs 95% 0.791; 0.850].

### Statistical analysis

For the statistical analyses, we used the package SPSS v. 26 for the verification of the univariate and multivariate hypotheses, the exploratory factor analysis (EFA) with Maximum Likelihood (ML) and Promax rotation, the assessment of internal consistency through Cronbach’s raw coefficient, and the assessment of the significance of correlation coefficients with bootstrap CIs in order to test the tool’s convergent validity; to calculate McDonald’s coefficient, we used the JASP 0.12.2 software, and IBM Amos Graphics 18 for the Confirmatory Factor Analysis (CFA) extraction method. The verification of the assumptions of univariate and multivariate normality has been conducted using the procedure for the standardization of the variables, erasing the outlier cases with values greater than 3; secondly, after calculating the Mahalanobis Distance, eliminating the multivariate outlier cases with D^2^ greater than the critical value, calculated by considering chi-square as the reference distribution (*p* < 0.001) with degrees of liberty equal to the number of variables. VIF values and Durbin–Watson test were considered to check for co-linearity and autocorrelation. To test the adequacy of the CFA model, as suggested by technical literature (Teo, 2010), Chi-square, CFI (*Comparative Fit Index*), TLI (*Tucker-Lewis Index*) and RMSEA (*Root-Mean-Square Error of Approximation*) were used as relevant fit indicators, with CFI and TLI > 0.95 and RMSEA < 0.06 as excellent model fit indicators (Yu, 2002).

The factorial structure of the *Fear of Pain Questionnaire* was tested for measurement invariance by gender. As a result, four layered models were evaluated, each with greater degrees of restriction (Manganelli et al., 2019): the base model examined configural invariance and permitted free estimate of all parameters for each group. The metric (weak) invariance model, which was layered within the configural model, adds the constraint of invariant factor loadings between groups to the configural model. The scalar (strong) invariance model, which was layered within the second model, adds the invariant items’ intercept constraint to the comparison groups. Finally, strict invariance was assessed by comparing the scalar model against a model that additionally required residuals to be identical across groups. We concentrated on comparing the CFI, TLI, and RMSEA indices because the Chi-square indices are sensitive to sample size. A variation of these indices more than 0.01 was used as a criteria to rule out the more restrictive model’s invariance and accept the more parsimonious model (Cheung and Rensvold, 2002). The group mean differences in latent variables were examined once the strict invariance was confirmed.

Convergent validity was determined by comparing the correlations between the short form of *Fear of Pain Questionnaire-III* factors and the factors that make up PCS, LCBS, COPE-NVI.

For the discriminant validity of the tool a statistical power analysis was performed for the sample size estimation. The effect size (ES) in this study was 0.30, considered to be medium using Cohen’s (1988) criteria. With an alpha err prob. = 0.05 and power (1 − β err prob) = 0.95, the projected sample size needed with this effect size (G*Power 3.1) was approximately *n* = 36 for this simple between/within group comparison.

In order to compare the perceived fear of pain in the groups before and after the vision of the videos, a two-way repeated measures ANOVA was run with two independent variables (group, video) and one dependent variable (fear of pain). The primary purpose is to understand if there is an interaction between these two factors on the dependent variable. As the number of participants in the groups was balanced, in order to determine the interaction between the variables, Pillai’s criterion was used instead of Wilks’ Lambda as it is more robust to unequal covariance matrices (Olson, 1976). Following Cohen (1988), partial Eta squared (ηp^2^) was the measure used to assess effect size (0.01 = small, 0.06 = medium, 0.13 = large). The level of significance was set at *p* < 0.05, while for the testing of multiple univariate interaction effects a Bonferroni adjustment was introduced by dividing the declared level of statistical significance by the number of dependent variables: *p* < 0.025 (i.e., *p* < 0.05 ÷ 2). As a statistically significant interaction was found, a follow-up investigation proceeded with the computation of simple effects tests, in order to reveal the degree to which one factor was differentially effective at each level of the second factor.

## Results

The calculation of the Mardia Index (average of the squares of the Mahalanobis Distances) produced a coefficient (976.23) lower than the limit value (1.123), confirming the assumptions of multivariate normality. Low co-linearity was indicated by the low VIF values (Variance Inflation Factor) < 2 and high tolerance values > 0.60. For verification of the assumptions on the residuals, the average between the standardized and raw residuals was equal to 0; the Durbin–Watson test had a value of 1.756 and was therefore indicative of the absence of autocorrelation.

The evaluation of the metric properties of the scale was conducted through a confirming analysis (CFA) designed to test the goodness of the three-dimensional model adopted by McNeil and Rainwater (1998). The averages and standard deviations for the single items are reported in the following Table 1.

**Table 1:** Descriptive statistics of the Italian Fear of Pain Questionnaire (FPQ-III).

**Item**	**M**	***SD***	**Bootstrap CI 95%**	**Kurtosis**	**Skewness**
Item 1	3.72	1.01	(3.64 to 3.80)	−0.110	−0.602
Item 2	1.96	0.95	(1.88 to 2.03)	0.081	0.858
Item 3	2.20	1.07	(2.11 to 2.29)	−0.277	0.667
Item 4	3.43	1.09	(3.34 to 3.51)	−0.699	−0.303
Item 5	3.56	1.08	(3.47 to 3.64)	−0.346	−0.560
Item 6	3.71	1.02	(3.63 to 3.80)	−0.020	-0.679
Item 7	2.44	1.05	(2.35 to 2.53)	−0.285	0.530
Item 8	2.20	1.23	(2.11 to 2.30)	−0.515	0.725
Item 9	3.43	1.02	(3.34 to 3.51)	−0.509	−0.313
Item 10	3.29	1.02	(3.21 to 3.37)	−0.454	−0.257
Item 11	2.27	1.19	(2.18 to 2.37)	−0.608	0.638
Item 12	2.21	1.06	(2.13 to 2.30)	−0.335	0.634
Item 13	4.32	1.00	(4.23 to 4.39)	1.608	−1.490
Item 14	2.17	1.14	(2.07 to 2.26)	−0.243	0.771
Item 15	3.08	1.13	(2.99 to 3.17)	−0.885	−0.081
Item 16	3.75	1.04	(3.66 to 3.83)	−0.342	−0.534
Item 17	3.06	1.21	(2.97 to 3.16)	−0.957	−0.132
Item 18	3.25	1.08	(3.16 to 3.33)	−0.713	−0.076
Item 19	2.11	1.06	(2.01 to 2.19)	−0.295	0.748
Item 20	3.32	1.09	(3.23 to 3.41)	−0.621	−0.244
Item 21	2.87	1.14	(2.78 to 2.97)	−0.933	−0.046
Item 22	2.40	1.13	(2.31 to 2.49)	−0.857	0.321
Item 23	1.81	1.01	(1.74 to 1.89)	0.411	1.117
Item 24	1.77	0.97	(1.69 to 1.86)	0.745	1.204
Item 25	4.64	0.66	(4.59 to 4.69)	1.139	−1.592
Item 26	3.15	1.19	(3.04 to 3.24)	−0.832	−0.279
Item 27	3.07	1.06	(2.98 to 3.16)	−0.644	−0.220
Item 28	2.14	1.05	(2.06 to 2.24)	−0.233	0.722
Item 29	2.64	1.12	(2.55 to 2.73)	−0.783	0.228
Item 30	2.20	1.00	(2.12 to 2.28)	−0.226	0.538

M, Mean; SD, Standard Deviation; CI, Confidence Interval. *N* = 708.

**Figure 1:**
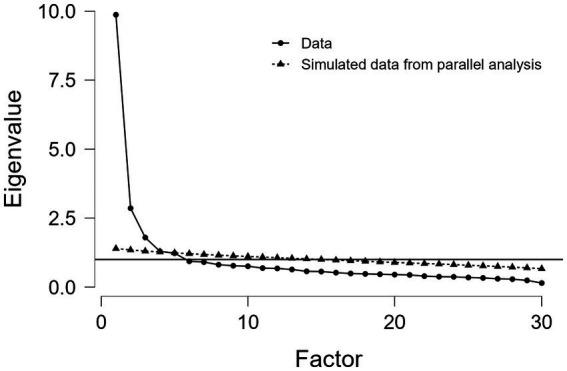
Scree plot.

In order to examine the validity of a 30-item scale, a confirmatory factor analysis was performed. The results obtained by considering three factors and 30 items did not show a good fit to the data: CFI = 0.756; TLI = 0.736; NNFI = 0.736; NFI = 0.724; RMSEA = 0.093; RMSEA 90% 0.90–0.0597; value of *p* = 4.38e − 9. Therefore, the existence of a lower number of items or different factor structure was verified by performing an EFA with ML on half of the sample (selected by randomization) and CFA on the remaining portion. Considering Promax as rotation method and Cattell’s scree test indications (that three main factors lay above the debris), 26 items resulted in the factor loadings Structure Matrix. Factor loadings indicated the elimination of the items 9, 16, 27, 29 arriving at a final number of 26 items. Kaiser–Meyer–Olkin (KMO) index score was 0.928, Bartlett’s test *p* < 0.001; Chi-squared Test < 0.001; RMSEA = 0.055, RMSEA 90% confidence 0.04–na; TLI = 0.66. Figure 1 shows the scree plot while loadings pattern matrix was reported in Table 2.

**Table 2:** Loadings pattern matrix EFA.

Factor loadings				
	Factor 1	Factor 2	Factor 3	Uniqueness
D17	0.812			0.454
D21	0.694			0.412
D14	0.685			0.530
D08	0.663			0.593
D11	0.660			0.563
D15	0.637			0.580
D20	0.636			0.519
D26	0.506			0.665
D18	0.407			0.770
D24		0.816		0.437
D23		0.765		0.565
D28		0.590		0.598
D02		0.546		0.637
D19		0.497		0.543
D07		0.496		0.672
D03		0.491		0.664
D30		0.456		0.595
D12		0.410		0.508
D22		0.405		0.512
D06			1.030	0.211
D05			1.004	0.168
D13			0.661	0.581
D04			0.618	0.557
D01			0.530	0.685
D10			0.480	0.600
D25			0.425	0.752
D09				0.611
D16				0.786
D27				0.724
D29				0.687

Applied rotation method is promax.

Subsequently, the items 1, 5, 12, 14, 15, 18, 19, 20, 22, 23, 25, 26, and 30 were removed because they were found to damage the fit between the model and the covariance structure. Through the omission of these 13 items the following fit values were reached: Kaiser–Meyer–Olkin (KMO) index score was 0.858, Chi-squared Test < 0.001; RMSEA = 0.063; RMSEA 90% 0.063–0.084; TLI = 0.950.

Table 3 shows the final pattern matrix with saturations on the three identified factors, McDonald’s ω and Cronbach’s Alpha values, Guttman Split-Half Coefficients, Corrected item/total correlations, factor intercorrelations; while in Table 4 the factorial interrelationships are reported.

**Table 3:** Pattern matrix EFA (13 items).

	Severe pain	Medical pain	Minor pain
Item 06	**0.809**	−0.021	−0.089
Item 13	**0.737**	0.011	−0.112
Item 04	**0.700**	−0.008	0.053
Item 10	**0.617**	−0.035	0.130
Item 08	−0.063	**0.879**	−0.046
Item 11	−0.086	**0.865**	0.018
Item 17	0.212	**0.552**	0.025
Item 21	0.258	**0.430**	0.182
Item 24	−0.212	−0.012	**0.795**
Item 02	0.092	−0.010	**0.606**
Item 03	0.149	−0.059	**0.603**
Item 28	−0.069	0.058	**0.574**
Item 07	0.122	0.026	**0.493**
*α*	0.796	0.804	0.751
ω	0.796	0.804	0.752
*λ6*	0.750	0.785	0.723
r*	0.61	0.62	0.65

Extraction Method, Maximum Likelihood; Rotation Method, Promax with Kaiser Normalization; Rotation converged in 5 iterations. Values above 0.4 are given in bold face. α, Cronbach’s alpha; ω, McDonald’s omega; λ6, Gutmann’s lamda; r*, average inter-item correlation.

**Table 4:** Factor inter-correlations.

	Severe pain	Medical pain	Minor pain
Severe pain	1		
Medical pain	0.478^**^	1	
Minor pain	0.532^**^	0.605^**^	1

**Correlation is significant at the 0.01 level (two-tailed).

**Figure 2:**
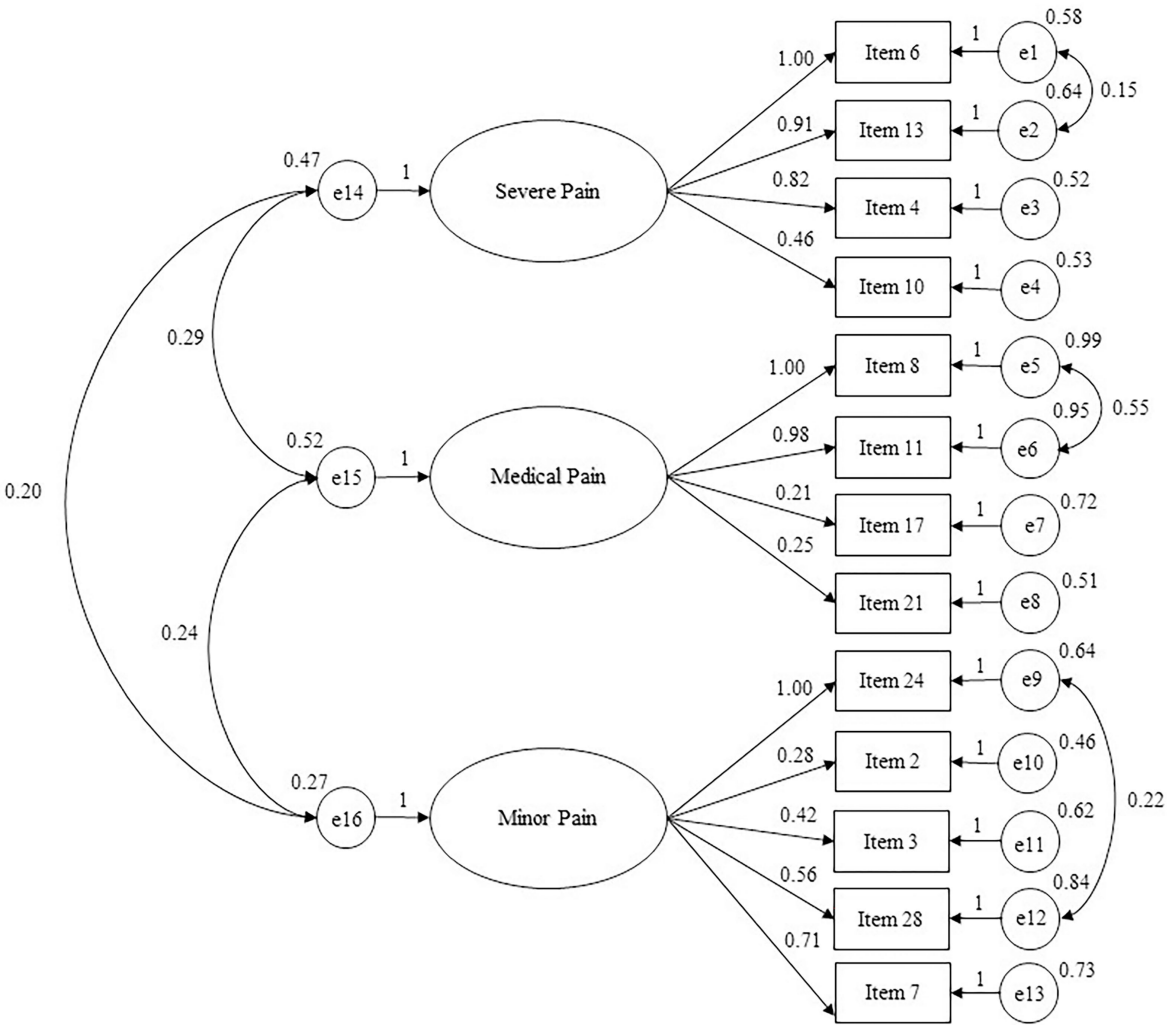
Path diagram of the confirmatory analysis concerning FPQ-III short form (13 items). χ^2^/df = 2.51; RMSEA = 0.046; RMSEA 90% CI = 0.037–0.056; GFI = 0.969; TLI = 0.962; CFI = 0.971; NFI = 0.953.

The confirmatory factor analysis performed with the split sample consisting of 354 participants confirmed that the model with three related factors and 13 items presented overall good indices of fit to data (see Figure 2): Chi-square = 148.092; χ^2^/df = 2.51; CFI = 0.971; TLI = 0.962; RMSEA = 0.046 and RMSEA 90% CI [0.037–0.056]. The first factor measures *Severe Pain Jealousy* (4 items); the second factor measures *Medical Pain* (4 items); the third factor measures *Minor Pain* (5 items).

The following Table 5 shows item statistics. All of the items showed some ceiling effects, and these ranged from 11.4% (item 10) to 58.3% (item 13). Item 13, which is the one that aroused the most fear of pain, had a mean score of 4.29, whereas item 24, which is the one that aroused the least fear of pain, had a mean score of 1.77. The overall mean score was 35.71 (13–65) with a *SD* of 8.46.

**Table 5:** The item statistics of FPQ-III short form.

**Response**	**Item 6**	**Item 13**	**Item 4**	**Item 10**	**Response**	**Item 8**	**Item 11**	**Item 17**	**Item 21**	**Response**	**Item 24**	**Item 2**	**Item 3**	**Item 28**	**Item 7**	**Overall**
Not at all (ceiling effect)					Not at all (floor effect)					Not at all (ceiling effect)						
1	2.8	2.4	4.2	4.4	1	38.3	32.5	11.7	13.7	1	49.9	35.9	29.7	32.6	19.5	
2	10.6	4.4	16.1	16.9	2	25.1	30.2	21.3	26.4	2	31.4	40.5	35.2	37.1	39.3	
3	22.7	12.1	27.1	33.5	3	18.9	18.5	25.1	26.1	3	11.9	14.1	21.8	17.8	25.3	
4	41.2	21.9	34.6	33.7	4	10.7	13.4	27.1	26.3	4	5.7	7.8	10.3	10.0	11.9	
5	22.6	58.3	17.9	11.4	5	6.1	5.4	12.1	6.6	5	1.3	0.8	3.1	2.4	4.1	
Extreme (floor effect)					Extreme (ceiling effect)					Extreme (floor effect)						
Mean	3.70	4.29	3.46	3.31	Mean	2.20	2.29	3.09	2.86	Mean	1.77	1.97	2.22	2.12	2.42	35.71
Standard deviation	1.02	1.01	1.09	1.02	Standard deviation	1.23	1.20	1.22	1.15	Standard deviation	0.95	0.95	1.07	1.05	1.06	8.46
Skewness	−0.62	−1.43	−0.35	−0.25	Skewness	0.75	0.64	−0.13	0.27	Skewness	1.22	0.85	0.56	0.77	0.54	0.29
Kurtosis	−0.15	1.41	−0.62	−0.47	Kurtosis	−0.48	−0.61	−0.94	−0.92	Kurtosis	0.95	0.15	−0.29	−0.12	−0.27	−0.30
Alpha (if item dropped)	0.84	0.85	0.84	0.84	Alpha (if item dropped)	0.84	0.84	0.84	0.84	Alpha (if item dropped)	0.85	0.84	0.84	0.85	0.84	
Item-total correlation	0.48	0.44	0.53	0.53	Item-total correlation	0.53	0.55	0.53	0.62	Item-total correlation	0.44	0.53	0.52	0.46	0.49	

Item numbering corresponds to that of the original McNeil and Rainwater (1998) scale.

Furthermore, the measurement invariance of the factorial structure of the FPQ-III by gender was assessed. Four nested models with increasing degrees of restriction were tested. Table 6 shows the goodness-of-fit indices of the multidimensional model by gender and nested models of invariance in ascending order of restriction level. Results showed that the FPQ-III had strict invariance across gender and that the fit of the three-dimensional model for males and females was excellent.

**Table 6:** Tested models and goodness-of-fit indices.

	χ^2^	*df*	Δχ^2^	Δ*df*	CFI	TLI	RMSEA	ΔCFI	ΔTLI	ΔRMSEA
**Models in each group**										
**Gender**										
Male	148.302	59			0.971	0.962	0.046			
Female	95.572	59			0.976	0.968	0.041			
**Gender**										
Configural	232.222^*^	118	-	-	0.963	0.951	0.052	-	-	-
Metric	243.633^*^	128	11.411	10	0.963	0.954	0.051	0.000	0.003	−0.001
Scalar	262.888^*^	136	19.255	8	0.960	0.954	0.051	−0.003	0.000	0.000
Strict	289.239^*^	154	26.351	18	0.956	0.956	0.050	−0.004	.002	−0.001

df, degrees of freedom; χ^2^, Chi square; Δχ^2^, difference in Chi square; Δdf, difference in degrees of freedom; CFI, comparative fit index; TLI, Tucker-Lewis index; RMSEA, root mean square error of approximation; ΔCFI, difference in comparative fit index; ΔTLI, difference in Tucker-Lewis index; ΔRMSEA, difference in root mean square error of approximation.

* *p* < 0.001.

These results indicate that the latent means can be compared by gender. The latent mean values were fixed to zero for females and, as can be seen in the following Table 7, males showed lower latent means of fear of pain in this study.

**Table 7:** Gender latent mean values.

Variable	Factor	Mean	*SE*	CR	*P*
Gender (male)^*^	Severe Pain	−0.98	0.13	−7.42	<0.001
	Medical Pain	−0.89	0.10	−9.25	<0.001
	Minor Pain	−0.82	0.11	−7.08	<0.001

SE, standard error; CR, critical ratio.

* Reference variable is female.

A further sample was used for convergent validity testing: correlations between the short form of FPQ-III, PCS, LBCS, and COPE-NVI were performed by administering a sample of 292 people, 113 males (38.7%) and 179 females (61.3%), M_age_ 24.46, and *SD* = 7.39. In relation to the hypotheses stated with regard to these associations, as shown in the following Table 8, the results have substantially confirmed the directions assumed. More specifically, the robust association with catastrophism (0.38^**^) and brooding thinking (0.39**) was found. Correlations with the other scales were congruent with what was expected, but still negligible in terms of effect size (<0.20). The association with external locus of control was positive (0.22^**^) while it was negative with respect to internally projected behavioral locus of control (−0.12^*^). Avoidance coping and transcendent reliance coping showed positive (albeit slight) relationships with fear of pain, 0.19^**^ and 0.13^*^, respectively.

**Table 8:** Bivariate correlations between Brief FPQ-III, PCS, LCBS, and COPE-NVI.

	1	2	3	4	5	6	7	8	9	10	11	12	13	14	15
FPQ (total score)	1														
Severe Pain (FPQ)	0.841^**^	1													
Medical Pain (FPQ)	0.775^**^	0.427^**^	1												
Minor Pain (FPQ)	0.811^**^	0.554^**^	0.471^**^	1											
PCS (total score)	0.385^**^	0.300^**^	0.314^**^	0.324^**^	1										
Helplessness (PCS)	0.315^**^	0.237^**^	0.251^**^	0.282^**^	0.918^**^	1									
Rumination (PCS)	0.395^**^	0.315^**^	0.321^**^	0.323^**^	0.913^**^	0.720^**^	1								
Magnification (PCS)	0.284^**^	0.226^**^	0.247^**^	0.215^**^	0.745^**^	0.568^**^	0.604^**^	1							
Internal control (LCBS)	–0.123^*^	–0.073	–0.107	–0.125^*^	–0.171^**^	–0.214^**^	–0.115	–0.080	1						
External control (LCBS)	0.220^**^	0.119^*^	0.230^**^	0.197^**^	0.425^**^	0.448^**^	0.312^**^	0.342^**^	–0.364^**^	1					
Social support (COPE)	0.196^**^	0.107	0.205^**^	0.175^**^	0.340^**^	0.300^**^	0.299^**^	0.307^**^	–0.084	0.194^**^	1				
Avoidance (COPE)	0.192^**^	0.086	0.177^**^	0.222^**^	0.361^**^	0.375^**^	0.257^**^	0.326^**^	–0.143^*^	0.402^**^	0.176^**^	1			
Positive attitude (COPE)	0.041	0.096	0.001	–0.011	–0.042	–0.115^*^	0.006	0.056	0.333^**^	–0.077	0.115	0.099	1		
Problem orient. (COPE)	0.033	–0.006	0.044	0.051	–0.026	–0.107	0.029	0.065	0.366^**^	–0.175^**^	0.213^**^	–0.015	0.653^**^	1	
Trascendent orient. (COPE)	0.131^*^	0.092	0.141^*^	0.085	0.070	0.081	0.069	0.002	–0.043	0.046	0.146^*^	–0.215^**^	–0.080	0.026	1

**Correlation is significant at the 0.01 level (two-tailed). *Correlation is significant at the 0.05 level (two-tailed).

In Table 9 the internal reliabilities of the two samples are shown comparatively with their confidence intervals. McDonald’s ω and Alpha coefficients for these convergent administrations ranged from 0.77 to 0.89 (*Severe Pain*), from 0.80 to 0.83 (*Medical Pain*), from 0.70 to 0.79 (*Minor Pain*), respectively.

**Table 9:** Internal reliabilities of the two samples.

	Sample 1 (*n* = 354)				Sample 2 (*n* = 292)		
	*α*	C.I.	ω	C.I.	*α*	C.I.	ω	C.I.
Severe pain	0.80	[0.77, 0.82]	0.79	[0.77, 0.82]	0.87	[0.84, 0.89]	0.87	[0.84, 0.89]
Medical pain	0.80	[0.78, 0.83]	0.80	[0.78, 0.83]	0.80	[0.76, 0.83]	0.80	[0.76, 0.84]
Minor pain	0.75	[0.72, 0.78]	0.75	[0.72, 0.78]	0.74	[0.70, 0.79]	0.75	[0.70, 0.79]

α, Cronbach’s alpha; ω, McDonald’s omega; C.I., 95% Confidence Interval.

The following Table 10 reports the English and Italian versions of the short form of FPQ-III, and the grouping of the items on respective factors.

**Table 10:** Fear of Pain Questionnaire (FPQ-III short form).

**English version**	**Italian version**
**INSTRUCTIONS**	**ISTRUZIONI**
The statements below describe painful experiences. Please read each statement and think about how scared you are of experiencing the pain associated with each statement. If you have never experienced the pain described in any of the statements, please respond based on how much fear you would expect to feel if you were to have such an experience. Indicate, for each statement below, the corresponding number, from 1 (not at all) to 5 (extreme), for the level of fear of pain you would experience in relation to each event described.	Le affermazioni di seguito elencate descrivono esperienze dolorose. Per favore legga ogni affermazione e pensi a quanto è spaventato dal vivere il dolore associato a ciascuna affermazione. Se non ha mai sperimentato il dolore descritto in una delle affermazioni, risponda in base a quanta paura si aspetterebbe di provare se le capitasse di vivere una tale esperienza. Indichi, per ogni affermazione di seguito riportata, il numero corrispondente, from 1 (not at all) to 5 (extreme), al livello di paura del dolore che proverebbe in relazione a ciascun evento descritto.
I FEAR the PAIN associated with:	Ho PAURA del DOLORE associato a:
1. Breaking your leg (SEV).	1. Rompermi una gamba.
2. Having a blood sample drawn with a hypodermic needle (MEP).	2. Il prelievo del sangue effettuato con l’ago della siringa.
3. Getting strong soap in both your eyes while bathing or showering (MIP).	3. Ad un sapone irritante che ti finisce negli occhi mentre ti fai la doccia o il bagno.
4. Breaking your neck (SEV).	4. Rompermi il collo.
5. Receiving an injection in your arm (MEP).	5. Ricevere un’iniezione sul braccio.
6. Biting your tongue while eating (MIP).	6. Mordermi la lingua mentre mangio.
7. Having a heavy object hit you in the Head (SEV).	7. Essere colpito sulla testa da un oggetto pesante.
8. Receiving an injection in your mouth (MEP).	8. Ricevere un’iniezione nella bocca.
9. Cutting your tongue licking an envelope (MIP).	9. Tagliarmi la lingua mentre lecco una busta.
10. Falling down a flight of concrete stairs (SEV).	10. Cadere rovinosamente dalle scale.
11. Having a foot doctor remove a wart from your foot with a sharp instrument (MEP).	11. Il podologo ti rimuove una verruca dal piede con uno strumento affilato.
12. Having sand or dust blow into your Eyes (MIP).	12. Sabbia o polvere volata negli occhi.
13. Hitting a sensitive bone in your elbow—your “funny bone” (MIP).	13. Sbattere la parte più sensibile del gomito.

SEV, Severe pain; MEP, Medical pain; MIP, Minor pain.

As far as the scoring of the instrument is concerned, the 13 items in total are distributed over three factors. Every item has a scoring range from 1 to 5. The scoring calculation produces, through a summation of the scores of the component items, separate measurements for each factor, and a total score that ranges from 13 to 65.

### Assessment of discriminant validity through the administration of fear eliciting videos

In order to compare the perceived fear of pain in each of the three subscales (severe, medical, and minor), before and after the vision of videos eliciting fear of pain, a two-way repeated measures ANOVA was run with two independent variables (group, video) and one dependent variable (fear of pain). A residual analysis was performed to test for the assumptions of the two-way repeated measures ANOVA. Scale reliability measures, also showed for this additional sample good coefficients: *Severe Pain*, Alpha 0.825 CI 95% [0.770; 0.869] and McDonald’s ω 0.825 CI 95% [0.770; 0.871]; *Medical Pain*, Alpha 0.821 CI 95% [0.765; 0.866] and McDonald’s ω 0.844 CI 95% [0.801; 0.882]; *Minor Pain*, Alpha 0.744 CI 95% [0.667; 0.806] and McDonald’s ω 0.752 CI 95% [0.696; 0.805]; *total Fear of Pain*, Alpha 0.843 CI 95% [0.799; 0.879] and McDonald’s ω 0.847 CI 95% [0.796; 0.880].

**Figure 3:**
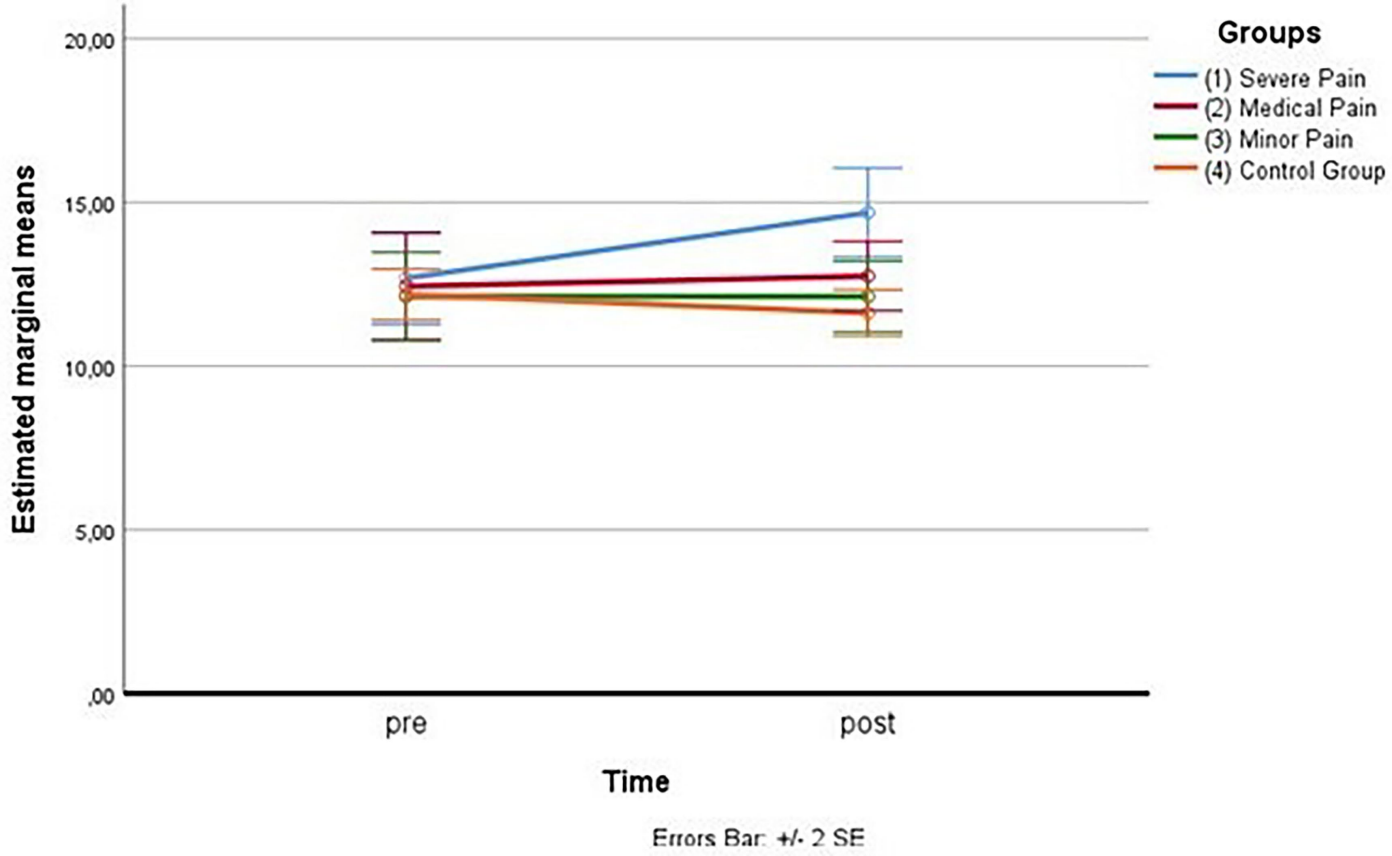
Estimated marginal means for the fear of severe pain.

As for the first measurement subscale (fear of severe pain), the outliers were assessed by inspection of a boxplot; normality was assessed using Shapiro–Wilk’s normality. Mauchly’s test of sphericity indicated that the assumption of sphericity was violated for the two-way interaction, χ^2^(5) = 11.69, *p* = 0.040, so the adaptation of Greenhouse–Geisser was considered. There was a statistically significant interaction between the group and time on fear of severe pain, *F*(2.005,30.072) = 16.329, *p* < 0.005, partial η^2^ = 0.521. (See Figure 3; Table 11 below.)

**Table 11:** Fear of severe pain means by comparing groups and time.

Group	Time	Mean	Stand. error	Confidence Interval 95%	
				Lower bound	Upper bound
1	Pre	12.688	0.705	11.184	14.191
	Post	14.685	0.681	13.235	16.140
2	Pre	12.438	0.811	10.708	14.167
	Post	12.750	0.528	11.624	13.876
3	Pre	12.125	0.676	10.684	13.566
	Post	12.125	0.539	10.976	13.274
4	Pre	12.188	0.390	11.357	13.018
	Post	11.625	0.352	10.875	12.375

**Figure 4:**
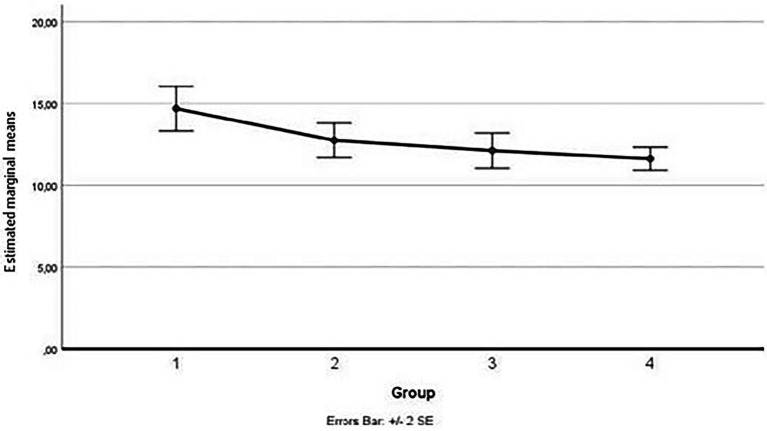
Estimated marginal means for the fear of severe pain (after the vision of videos).

Considering simple main effects, at the pre-test (before administration of videos to the participants), there was no significative difference regarding the fear of severe pain means between the groups, *F*(1.670,25.053) = 0.114, *p* > 0.05, partial η^2^ = 0.008; while at post-test, the main effect of group showed a statistically significant difference in fear of severe pain means: *F*(3, 45) = 5.580, *p* < 0.05, partial η^2^ = 0.271. A *post-hoc* analysis with a Bonferroni adjustment revealed that after the administration of the video soliciting fear of severe pain to the Group 1, participants in this Group reported fear of severe pain statistically significantly greater (M = 14.69 ± 2.73) compared to the Group 3 (M = 12.12 ± 2.16), *p* < 0.025, that had seen the video with content related to minor pain, and to the Group 4 (M = 11.62 ± 1.41), *p* < 0.025, consisting of the controls, but not with respect to Group 2 (M = 12.75 ± 2.11), *p* < 0.025, that had seen the video with content related to medical pain (see Figure 4).

**Figure 5:**
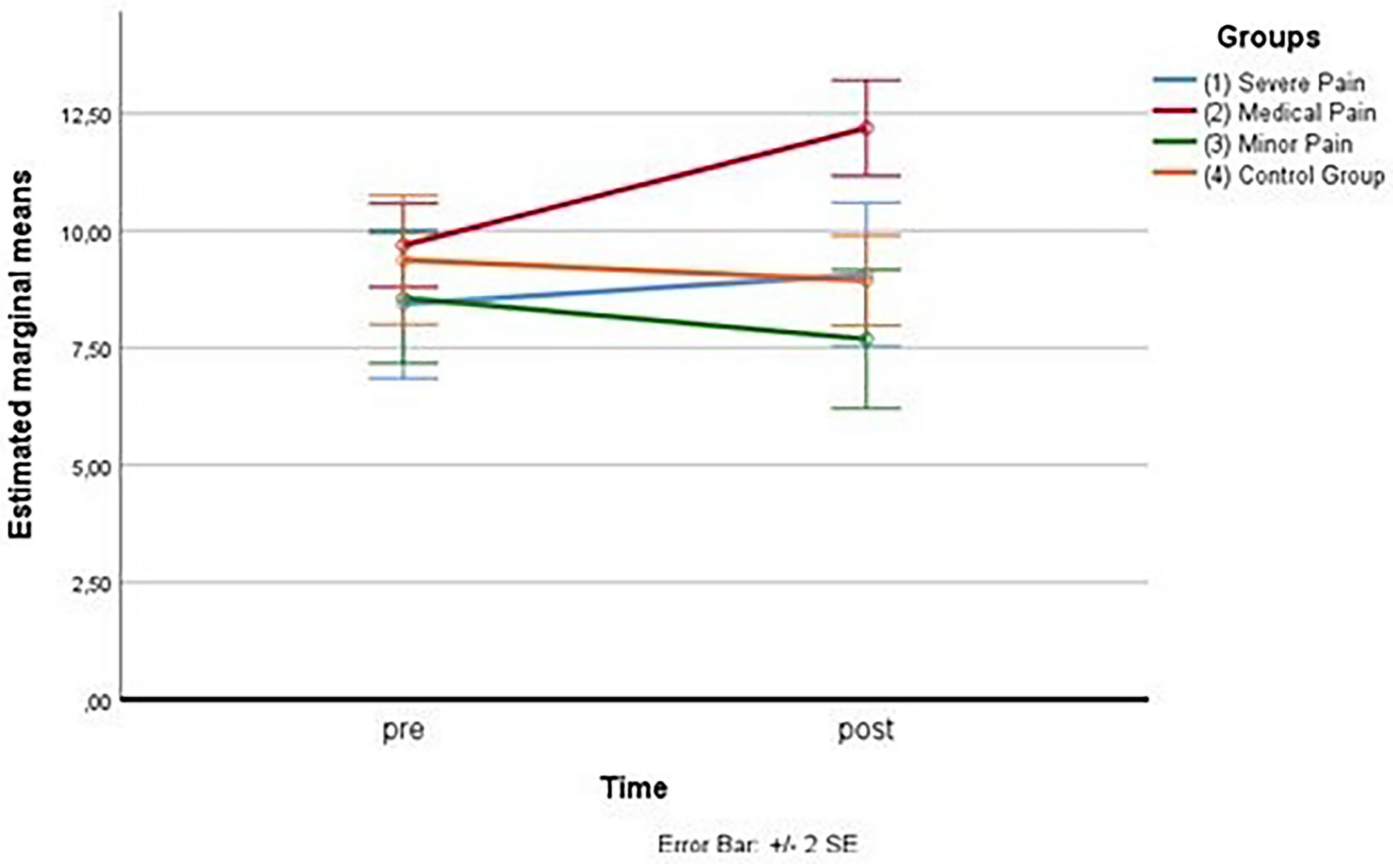
Estimated marginal means for the fear of medical pain.

As for the second measurement subscale (fear of medical pain), the outliers were assessed by inspection of a boxplot too; normality was assessed using Shapiro–Wilk’s normality. Mauchly’s test of sphericity indicated that the assumption of sphericity was not violated for the two-way interaction, χ^2^(5) = 9.83, *p* = 0.081. There was a statistically significant interaction between the group and time on fear of medical pain, *F*(3,45) = 46.696, *p* < 0.001, partial η^2^ = 0.757. (See Figure 5; Table 12 below.)

**Table 12:** Fear of medical pain means by comparing groups and time.

Group	Time	Mean	Stand. error	Confidence Interval 95%	
				Lower bound	Upper bound
1	Pre	8.438	0.796	6.742	10.133
	Post	9.063	0.766	7.429	10.696
2	Pre	9.688	0.445	8.740	10.635
	Post	12.188	0.510	11.100	13.275
3	Pre	8.563	0.695	7.081	10.044
	Post	7.688	0.740	6.110	9.265
4	Pre	9.375	0.688	7.908	10.842
	Post	8.938	0.478	7.918	9.957

**Figure 6:**
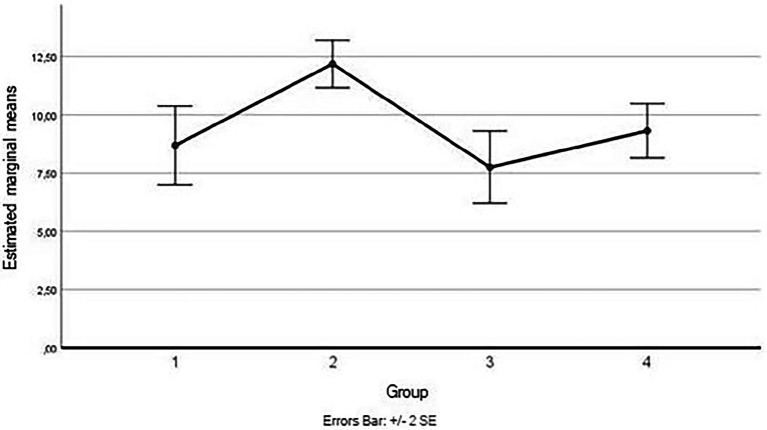
Estimated marginal means for the fear of medical pain (after the vision of videos).

Considering simple main effects, at the pre-test (before administration of videos to the participants), there was no significative difference regarding the fear of medical pain means between the groups, *F*(3,45) = 0.952, *p* > 0.05, partial η^2^ = 0.060; while at post-test, the main effect of group showed a statistically significant difference in fear of medical pain means: *F*(3, 45) = 9.057, *p* < 0.05, partial η^2^ = 0.376. A *post-hoc* analysis with a Bonferroni adjustment revealed that after the administration of the video soliciting fear of medical pain to the Group 2, participants in this Group reported fear of medical pain statistically significantly greater (M = 12.19 ± 2.04) compared to the Group 1 (M = 9.06 ± 3.07), *p* < 0.025, that had seen the video with content related to severe pain, to the Group 3 (M = 7.69 ± 2.96), *p* < 0.025, that had seen the video with content related to minor pain and to the Group 4 (M = 8.94 ± 1.91), *p* < 0.025, consisting of the controls (see Figure 6).

**Figure 7:**
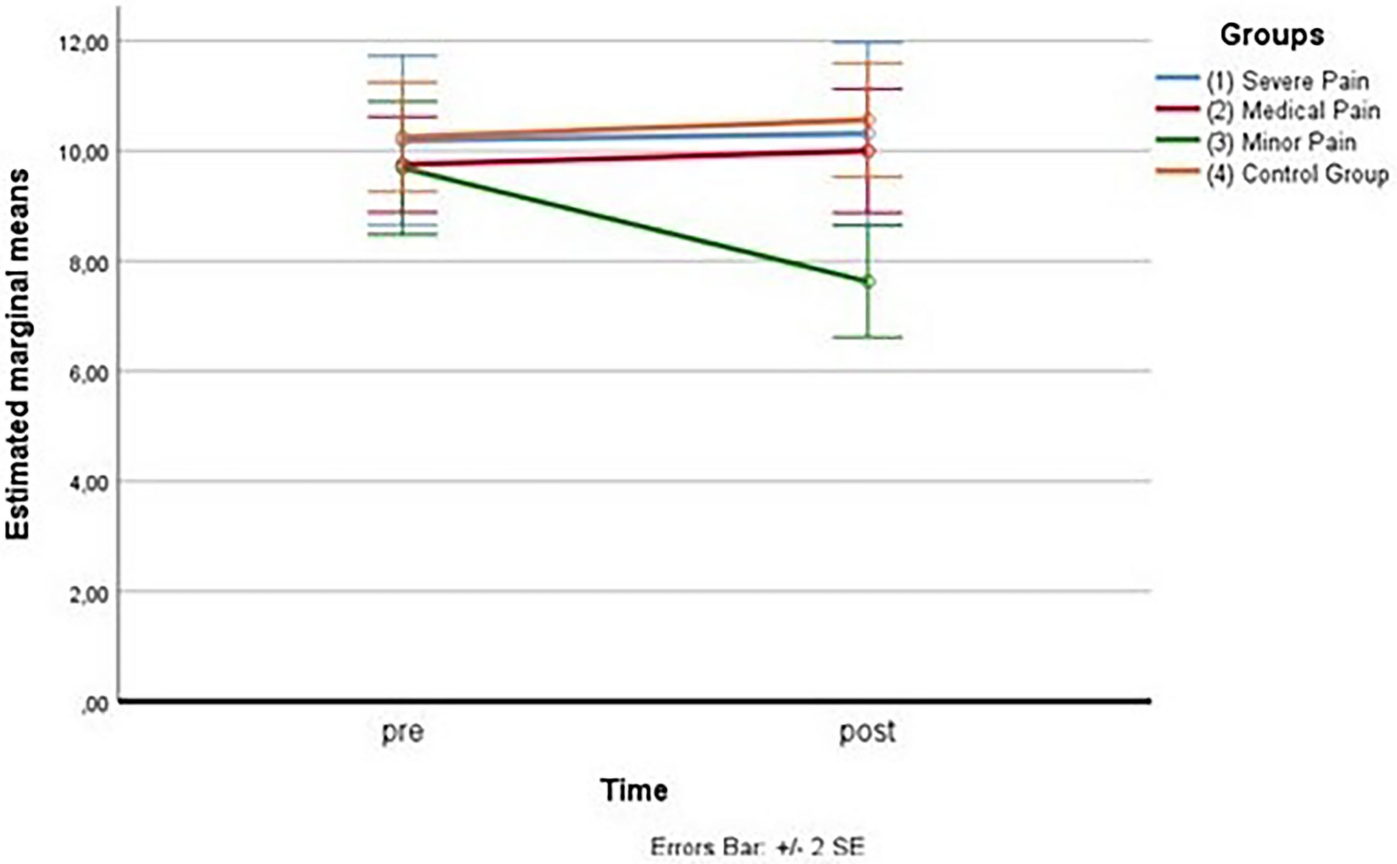
Estimated marginal means for the fear of minor pain.

As for the third measurement subscale (fear of minor pain), the outliers were assessed by inspection of a boxplot; normality was assessed using Shapiro–Wilk’s normality. Mauchly’s test of sphericity indicated that the assumption of sphericity was not violated for the two-way interaction, χ^2^(5) = 7.45, *p* = 0.1911. There was a statistically significant interaction between the group and time on fear of minor pain, *F*(3,45) = 13.928, *p* < 0.001, partial η^2^ = 0.481. (See Figure 7; Table 13 below.)

**Table 13:** Fear of minor pain means by comparing groups and time.

Group	Time	Mean	Stand. errors	Confidence interval 95%	
				Lower bound	Upper bound
1	Pre	10.188	0.770	8.545	11.830
	Post	10.313	0.830	8.543	12.082
2	Pre	9.750	0.433	8.827	10.673
	Post	10.000	0.563	8.801	11.199
3	Pre	9.688	0.604	8.401	10.974
	Post	7.625	0.507	6.544	8.706
4	Pre	10.250	0.496	9.193	11.307
	Post	10.563	0.516	9.462	11.663

**Figure 8:**
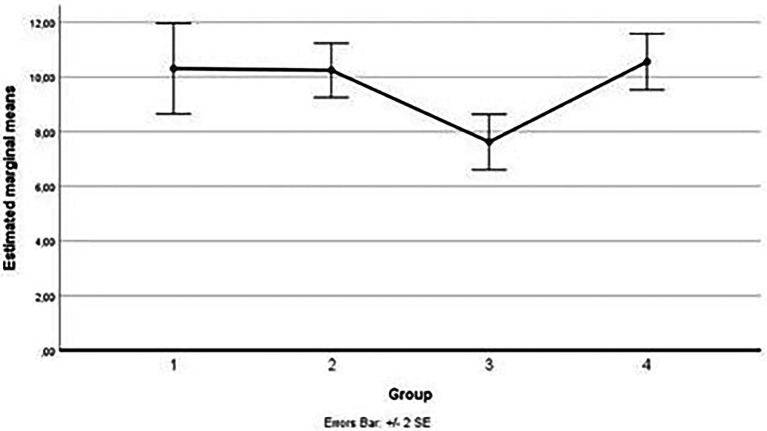
Estimated marginal means for the fear of pain (after the vision of videos).

Considering simple main effects, at the pre-test (before administration of videos to the participants), there was no significative difference regarding the fear of minor pain means between the groups, *F*(3,45) = 0.239, *p* > 0.05, partial η^2^ = 0.016; while at post-test, the main effect of group showed a statistically significant difference in fear of minor pain means: F(3, 45) = 5.659, *p* < 0.001, partial η^2^ = 0.274. A *post-hoc* analysis with a Bonferroni adjustment revealed that after the administration of the video soliciting fear of minor pain to the Group 3, participants in this Group reported fear of minor pain statistically significantly greater (M = 7.62 ± 2.03) compared to the Group 1 (M = 10.31 ± 3.32), *p* < 0.025, that had seen the video with content related to severe pain, to the Group 2 (M = 10.25 ± 1.98), *p* < 0.025, that had seen the video with content related to medical pain and to the Group 4 (M = 10.56 ± 2.06), *p* < 0.025, consisting of the controls (see Figure 8).

In conclusion, the evaluation of the effect induced through the administration to three groups of three videos with content aimed at soliciting fear of severe pain, medical pain and minor pain in each, respectively, showed that the instrument used was adequate to detect the different variations in fear of pain in the groups, obtained through the experimental manipulation carried out using the activating videos. The results showed that watching the videos with severe pain and medical pain content induced greater fear in the respective groups compared to the baseline measurement and compared to a control group; on the other hand, watching a video with minor pain content induced a subsequent lowering of the level of fear in the group, both compared to the other groups and to the preliminary baseline assessment.

## Discussion

The aim of this work was to present a validation study for an Italian short form of the *Fear of Pain Questionnaire (FPQ-III).* The analyses carried out led to the definition of a scale composed of a total of 13 items that converge separately on three factors (fear of severe pain, medical pain, and minor pain), showing an overall good fit of the model.

The first factor measures the person’s fear of receiving major damage on some part of the body as a result of events such as a disastrous fall downstairs, a broken limb, a head injury, or a broken neck. The convergent validity analysis indicated the significant positive associations with the sub-scales of catastrophism, namely brooding, magnification, helplessness, and with external causal attribution. The first relationship between brooding thought and fear of major painful harm is a confirmation of one of the main convergence hypotheses posed by this study. There is both a strand of studies in the literature that predictively links remorseful amplification to more intense pain perception (Burri et al., 2018; Priore et al., 2019; Diotaiuti et al., 2021; Häggman-Henrikson et al., 2021; Nunziato et al., 2021), and further empirical evidence reporting a direct effect of fear of pain on the level of actually perceived pain (Markfelder and Pauli, 2020; Luo et al., 2022). According to some scholars, fear of pain constitutes a stronger predictor of actually perceived pain than depressive catastrophic amplification (George et al., 2006; Hirsh et al., 2008). In each case, amplification orientation reported a significant association with the level of fear of pain in our study. An additional association was reported with the helplessness measure that is characterized by negative outcome expectancies and general, stable negative attributions ascribed to the actual pain condition. This is consistent with what has already been reported in the study by Samwel et al. (2006) where there was a robust correlation between helplessness and fear of pain. Feelings of helplessness may be an important consequence of the learning history of chronic pain patients, especially those with many years of pain. It refers to an attributional style, explaining negative events such as chronic pain and its consequences as uncontrollable, unpredictable, and unchangeable and generalizing these consequences to daily functioning (Samwel et al., 2007; Craner et al., 2016; Burland et al., 2019; Stensland, 2021). The association between fear of severe pain and external locus of control confirms findings in studies where lack of control over a totally external and unpredictable source of danger has been found to be associated with higher estimated potential harm (Heinze and Sleigh, 2003; Guszkowska, 2014; Musich et al., 2020).

The medical pain fear subscale reported significant associations not only with the catastrophizing measure but also with avoidance coping and external social support seeking. The first association is congruent with the literature where there is a recurrent association between fear of pain and avoidance coping (Fischerauer et al., 2018; Meulders, 2019; Buchmann et al., 2021). As suggested by van Vliet et al. (2018), the ability to avoid painful situations may paradoxically even increase the levels of fear elicited by such situations. The Fear Avoidance Model (FAM) currently represents a solid theoretical reference for understanding fear response behaviors generated by potentially painful situations (Crombez et al., 2012; Wideman et al., 2013). Maladaptive fear-avoidance behaviors include catastrophizing, anxiety/depression, and avoiding physical and social activities. These behaviors are linked to increased pain, disability, and sadness and are thought to be caused by a disorder that resembles kinesiophobia (Vlaeyen and Linton, 2000; Peeters et al., 2020). As a result, people perceive painful experiences as a warning of impending danger, injury, or a catastrophic medical ailment (catastrophizing), which causes them to dread pain and make actions associated to it. Increases in pain perception (hypervigilance) and the avoidance of pain-related movements are both consequences of elevated pain-related anxieties. Short-term reductions in pain and mental suffering may encourage avoidance behavior. Long-term avoidance and restriction of physical and social activity, on the other hand, may encourage physical deconditioning, accrue loss of social reinforcement, and eventually lead to a disuse syndrome (Nees and Becker, 2018). Contrarily, it is believed that no-fear confrontational pain reactions without catastrophizing and conditioned avoidance behavior help to prevent chronic pain and speed up healing, considering that physical exercise is considered an excellent tool for the treatment of chronic pain (Verbunt et al., 2003; Diotaiuti et al., 2022a). Coping oriented toward seeking external support also registered a positive association with fear of pain. The result finds an explanation in the disposition with low autonomy and limited action orientation of the person who preferentially seeks comfort and emotional support from others to cope with the strong fear of pain he or she is experiencing. The significant protective role of social support in mitigating the pressure of stress and perceived distress had already been emphasized in previous studies (Koivula et al., 2002; Zaza and Baine, 2002; Azimi et al., 2018). Complete and passive reliance on external support, however, can generate increased fear of pain when people who should provide emotional containment instead indirectly defer their anxieties and concerns about the unfolding of their relative or caretaker’s delicate situation.

In this regard, Neville et al. (2021) illustrated the situation of parents who had ended up increasing (by failing to contain their own anxieties and fears) in their children the fear of pain related to the medical care they were to receive. Beyond the non-functional response that support figures may have, evidence has emerged that illustrates a remarkable inhibitory effect of the presence of support figures on the formation of new fear associations for other cues, allowing the person to move with fewer learned fears, thus decreasing the activation of the threat response (Hornstein and Eisenberger, 2017). More recent models of pain coping emphasize the positive function of actively building individual resilience resources, where the focus of intervention should be on behavior change and sustaining, restoring or promoting the undertaking of personal goal-directed actions in the presence of pain (Goubert and Trompetter, 2017). Petter et al. (2014) demonstrated that changes in pain catastrophizing, which were indirectly connected to an induction of state mindfulness, were associated with lower levels of pain and improved pain tolerance. According to research, positive attitudes like optimism and acceptance coping may independently predict positive results from unfavorable personal traits and events (Smith and Zautra, 2008; Jackson et al., 2014; McCracken and Vowles, 2014).

The third measure of FPQ-III, related to fear of mild painful sources and conditions without major physical consequences, in addition to the correlations highlighted above in relation to medical pain, showed a significant inverse association with internal locus of control. In contrast to the fear of dangers and events relevant to physical safety or the outcome of medical manipulations or interventions, in the case of minor pain, the person perceives a wider sphere of control, which allows him or her a greater range of action or choice: to avoid certain situations, to better control processes, to take action on possible preventive measures. In any case, awareness of the limited duration of the condition predisposes the person to greater tolerance and forbearance, limiting the intensity of anticipatory fear associated with such conditions. However, in the literature, the role of internal locus of control in mitigating the experience of mild pain is reported in several studies (Bonafé et al., 2018; Löffler et al., 2018; Musich et al., 2020; González-Roldán et al., 2021).

Consistent with evidence reposted in the literature (Horn et al., 2014; Ramírez-Maestre and Esteve, 2014), analysis of invariance and related measures of latent averages, showed significantly higher levels of fear of pain in females, with particular note for the subscale of fear of pain attributable to medical treatment. However, this result differs from what was found in Vambheim and Øien (2017)’s previous study, in which the greatest gender difference was found in the severe pain factor, with the highest fear manifested by the female group. The authors attributed this difference to a higher level of fear of dying, fear of being disabled, overall anxiety, or existential anxiety, related to the consequences and implications that serious happenings might have effect on, for example, familial factors and caregiver responsibilities. Another explanation by the authors was related to gender role expectations of pain that influence males and females differently when confronted with reporting fear of pain. In this regard, the authors refer to the work of Wise et al. (2002) and Aslaksen et al. (2007) where it is shown that pain behavior is influenced by contextual factors, such as socialization, cultural expectations, and feminine and masculine gender role expectations. Therefore gender role stereotypes might have contributed to the findings of the higher fear of pain in females than in males. In relation to measures of fear of medical pain and minor pain in females, Fredrikson et al. (1996) had already reported that they have higher fear of injections, dentists, and injuries, compared to males; they worry, ruminate, and display more negative affectivity, and are more afraid of burning their fingers, cutting themselves while shaving, having a muscle cramp (minor pain), and having a wart removed by a doctor, compared to males. Courbalay et al. (2016) hypothesize a significant mediating role of neuroticism on the level of female fear of severe and medical pain. The increased sensitivity to medical pain in females is also reported by more recent studies such as Heft et al. (2007), Katanec et al. (2018), McLenon and Rogers (2019), and Labrenz et al. (2020). In addition, female seem to have a significantly higher perceived pain intensity than men, which could also explain these findings (Diotaiuti et al., 2022b). The test of measurement invariance with respect to gender in our study showed that the short version of the FPQ-III is an appropriate instrument for assessing fear of pain in males and females in the Italian context.

Discriminant assessment using the videos confirmed the adequacy of the scale in detecting changes in fear of pain in the groups obtained through the experimental manipulation. The results showed that watching the videos with severe pain and medical pain content induced greater fear in the respective groups compared to the baseline measurement and compared to a control group; on the other hand, watching a video with minor pain content induced (unexpectedly) a subsequent lowering of the level of fear in the group, both compared to the other groups and to the preliminary baseline assessment. This last result appears interesting and could be interpreted as an identificatory response with situations where the pain shown was not associated for the person with major or potentially irreversible consequences, such as in the case of serious accident or surgery. Observing others involved in painful but occasional, temporary and minor events may have elicited a feeling of potential self-efficacy and confidence in managing and overcoming the unpleasant situation, lowering the level of anticipatory fear associated with such situations.

## Conclusion

In the present study, the Italian Short Version of the Fear of Pain Questionnaire (FPQ-III) showed good psychometric properties. The three-factor model to measure the fear of severe, medical and minor dimensions of pain was confirmed. In addition, the measurement invariance of the scale across gender was established with an extended sample of participants. The values of internal consistency and convergent validity were good as well. A further discriminant assessment using experimental manipulation through administration of fear eliciting videos demonstrated that the instrument is suitable in detecting changes in fear of pain induced by changes in situational conditions. The scale in this short version is particularly suitable to quickly and efficiently collect information on the perceived intensity of such anticipatory fears that could also dysfunctionally affect the person. High medical fear, for example, can activate avoidance responses with negative repercussions for diagnostic monitoring, delaying any necessary interventions and therapeutic treatments. Excessive fear of severe pain (coupled with continued fears of accidents, serious injuries, disabling injuries, etc.) can lead the person to progressive self-limitation in movement and normal interaction with his or her surroundings, lowering his or her overall quality of life. Finally, excessive fear of frequent minor painful events closely associated with the sphere of daily life (cutting oneself, tripping, hitting an edge, having dust or stinging liquid in one’s eyes, etc.) may portend states of anxiety and difficulty in focusing on coordinating actions in personal space. It therefore appears important to assess the intensity and frequency of such anticipatory fears in order to put in place restraining interventions and functional support for the person. The Italian validation of the short version of the FPQ-III is therefore a useful and original contribution to the conduct of such an assessment, possibly associated with other more in-depth psychometric instruments.

## Data availability statement

The raw data supporting the conclusions of this article will be made available by the authors, without undue reservation.

## Ethics statement

The studies involving human participants were reviewed and approved by Institutional Review Board (IRB) of the University of Cassino and Southern Lazio. The participants provided their written informed consent to participate in this study.

## Author contributions

PD, SC, and SM designed the study, analyzed the data, and discussed the results. PD, AC, and EC drafted the manuscript. TS and AA revised the manuscript. All authors contributed to the article and approved the submitted version.

## Conflict of interest

The authors declare that the research was conducted in the absence of any commercial or financial relationships that could be construed as a potential conflict of interest.

## Publisher’s note

All claims expressed in this article are solely those of the authors and do not necessarily represent those of their affiliated organizations, or those of the publisher, the editors and the reviewers. Any product that may be evaluated in this article, or claim that may be made by its manufacturer, is not guaranteed or endorsed by the publisher.
